# The Mental Health Technology Assessment of Quality (MTAQ): Development of a novel quality assurance framework for digital mental health tools

**DOI:** 10.1177/20552076261428307

**Published:** 2026-02-24

**Authors:** Chris Attoe, Raul Szekely, Marta Ortega Vega, Emma Browne, Rebecca Thomas, Cho Wing Tiffany Kwan, Polina Altchouler, Anushka De, Nina Hariharan, Geraldine Clay, Sarah Odoi, Sean Cross, Andrew Doe

**Affiliations:** 1 4958 South London and Maudsley NHS Foundation Trust, London, UK; 2 4616 Institute of Psychiatry, Psychology, and Neuroscience, King's College London, London, UK; 3 3661 Royal Surrey NHS Foundation Trust, Guildford, UK; 4 School of Psychology, 3660 University of Surrey, Guildford, UK; 5 Wolfson Institute of Population Health, 4617 Queen Mary University of London, London, UK

**Keywords:** Digital mental health, quality assurance, user experience, data security, evidence

## Abstract

**Background:**

The rapid growth of digital mental health tools offers accessible support to a growing global population with mental health needs. However, concerns remain regarding their safety, efficacy, and overall quality, particularly for tools used outside formal healthcare settings. Limited regulation and non-specific quality frameworks exacerbate these issues, often overlooking key elements such as user experience and evidence-based practice.

**Objective:**

This study aimed to develop a comprehensive and pragmatic quality assurance framework for digital mental health tools, tailored to the needs of UK stakeholders.

**Methods:**

A sequential mixed-methods design informed the development of the framework. Participants included mental health service users, academic and clinical professionals, and digital health experts. Survey data (*N* = 309) were analysed descriptively to identify stakeholder priorities across three core domains: user experience, data, and evidence. These findings were enriched by qualitative data from focus groups (*N* = 101), analysed thematically using a deductive approach to refine and extend the framework.

**Results:**

Intuitive, accessible, user-centred design, strong data privacy and security measures, and robust evidence were identified as essential to quality assessment. These findings informed the development of MTAQ, which includes key domains and a structured process for evaluating tool quality.

**Conclusions:**

The MTAQ framework is among the first quality assurance frameworks specifically designed for digital mental health tools. By integrating research evidence with lived experience, clinical, academic, technical, and commercial input, this offers a user-informed, practical standard to guide the development, assessment, and improvement of digital mental health technologies.

## Introduction

With the increasing prevalence of mental health concerns, many consumers are turning to digital mental health tools due to their unique advantages of cost effectiveness and ready availability.^1–3^ However, there is a lack of regulation of those used outside healthcare. Frameworks have been proposed to establish agreed-upon standards, but these overlook key aspects that research and stakeholder perspectives indicate are important to mental health tool quality. This study addressed this issue by consulting mental health service users, academics and healthcare professionals, and digital health provision experts to establish which research-backed aspects of digital mental health tool quality are important across stakeholders and how a quality verification process can best meet their needs.

## Background

Improving global mental health has become an urgent priority in recent years. In 2019, an estimated 12.5% of the world's population, nearly one billion people, were living with a mental health condition,^4^ a figure that surged in the wake of the COVID-19 pandemic.^5^ Despite the growing prevalence of mental health issues, access to mental health services is limited, particularly in low- and middle-income countries.^6^ Digital mental health products such as smartphone apps, virtual reality, and chatbots are often presented as effective, scalable ways to bridge the gap between demand and capacity.^7–11^ These tools are accessible and affordable and provide additional benefits such as identifying problems in real time, reducing the financial burden of mental healthcare, circumventing stigma against help-seeking, and enhancing therapeutic outcomes.^12^

A multitude of digital mental health tools have been developed and launched in response to this potential. By 2021, there were an estimated 20,000 smartphone mental health apps,^13^ and this sector is projected to show a compound annual growth rate of 15.2% from 2024 to 2030.^14^

### Quality concerns

Despite their potential, there are concerns about the safety and efficacy of these digital mental health tools. A systematic review of 293 apps offering treatments for anxiety or depression showed that while 55% mentioned evidence-based frameworks in their app store descriptions, only 10 had published research supporting their effectiveness.^15^ Even more concerning, another systematic review^16^ found that 6 out of 82 apps for bipolar disorder, a condition with specific and distinct guidelines, contained inaccurate information. For example, one gave potentially dangerous advice to consume alcohol during a manic episode.

Even when validation has been performed, a lack of rigorous scientific evaluation often undermines the reliability of efficacy evidence.^17,18^ For instance, a meta-review of studies on mental health smartphone apps^19^ found that the few that evaluated effectiveness and efficacy had small sample sizes. Furthermore, where studies have shown digital tools to be effective in treating various mental health difficulties, these benefits commonly occur only compared to inactive controls,^20^ casting doubt on the importance of the app's mental health content to its effectiveness. Additionally, updates to these tools, which may be continuous if they utilise machine learning, mean that initial evaluations may not reflect the tool's current quality.

Concerns about quality are particularly stark for mental health tools used to support wellbeing outside of healthcare services. Due to long waiting lists for mental healthcare (e.g., reaching 1.2 million people in mid-2022 in the UK^21^), many people access digital mental health tools independently when experiencing mental health symptoms. Moreover, many people without diagnosable mental health conditions access them in pursuit of greater wellbeing. In response to these trends, the most commonly used mental health apps are targeted directly to consumers, providing self-help tools like mindfulness (e.g., Headspace, https://www.headspace.com/) and cognitive-behavioural exercises (e.g., Happify, https://www.happify.com/). However, unlike digital mental health tools used within healthcare,^22,23^ those marketed to consumers are entirely unregulated. This results in consumers not knowing which of the many tools available offers safe, high-quality support and developers lacking metrics against which to check their product's quality.

### Means of establishing quality

To address the challenges in evaluating digital mental health tools, one potential solution is for developers to adhere to established international^24^ and region-specific standards^25^ or frameworks that integrate these guidelines, such as the Organisation for the Review of Care and Health Apps (ORCHA).^26^ While not specific to digital mental health, ORCHA involves a structured assessment process that evaluates apps across different domains including data privacy, professional and clinical assurance, and user experience, and its reviews are embedded within many NHS (National Health Service, the publicly funded healthcare system of the UK) systems, facilitating integration into clinical pathways.^27–29^ These types of frameworks can therefore support both market access and user confidence. However, many regulatory and evaluative schemes focus on minimum baseline quality criteria, which may be insufficient for supporting more detailed assessment or guiding meaningful product improvement; in some cases, their complexity or lack of practical guidance can also make them difficult for developers to navigate.^30–34^ Furthermore, because these frameworks are often designed with consumers, clinicians, or procurement teams in mind, they tend to offer limited value to developers seeking structured, in-depth feedback to improve product quality.

An alternative approach involves developing a more robust framework specifically designed to evaluate the quality of digital mental health tools and provide actionable feedback to both developers and consumers. For example, frameworks such as the Mobile App Rating Scale (MARS)^35,36^ or the Enlight Suite^37^ set out comprehensive, multidimensional criteria for evaluating digital health apps. However, despite their potential, these frameworks have not gained widespread adoption among either developers or consumers within the mental health tool market. In addition, neither MARS nor Enlight Suite, nor existing industry standards, are specifically tailored to the unique aspects of mental health tools, such as the fidelity of interventions to evidence-based mental health treatments, the inclusion of mental health-specific risk management features (e.g., crisis support or symptom monitoring), and usability considerations for individuals with cognitive, emotional, or sensory difficulties. Omitting these factors can limit the frameworks’ rigour and overall effectiveness in improving the quality and safety of mental health digital tools.

Research indicates that quality in digital mental health tools encompasses a broad range of factors. For example, user experience (UX) and user interface (UI) emerged as key factors in a study analysing reviews of 106 mental health apps, where consumers valued apps with user-friendly interfaces, customisation options, and content variety.^38^ Bunyi and colleagues^39^ further highlight the importance of prioritising UX by ensuring accessibility for diverse, underserved, and vulnerable populations, including those with physical, sensory, and cognitive disabilities, to enhance usability and promote equitable access to mental health care. Additionally, providing a good UX is thought to be crucial for engagement, with intuitive and easily navigable interfaces recommended to improve usability and minimise technical issues.^40,41^

A further indicator of quality is a basis in *evidence*. As discussed above, many digital tools and interventions lack fidelity to evidence-based treatments, with rigorous testing often absent. Additionally, essential components such as human support are frequently missing from publicly available versions.^9^ This lack of transparency extends to the clinical evidence and technical implementation of apps, with only a small proportion of top-ranked apps providing published trial results, making it challenging for both patients and professionals to assess their credibility.^42^ Similarly, Borghouts et al.^41^ found that uncertainty about the credibility of the information, particularly regarding the evidence-based and the source, may impede user engagement and trust in digital mental health interventions. Furthermore, while approaches such as computerised cognitive-behavioural therapy have shown effectiveness for managing anxiety and depression in adolescents and young people, other digital interventions do not benefit from the same level of consistent evidence.^43,44^

Moreover, decision-makers in healthcare settings stress the importance of robust evidence before adopting and scaling digital health tools, with concerns that developers are sometimes unaware of the required levels of evidence needed for approval.^45^ However, when it comes to the type of evidence required, an expert workshop with researchers, clinicians, consumers, and policymakers highlighted that while randomised controlled trials are widely regarded as the “gold standard” and are generally valuable, they may not always be feasible or of the highest quality.^46^ Consequently, other forms of evidence, such as adherence data, consumer ratings, safety information, and expert clinical reviews, should also be incorporated to provide a more comprehensive evaluation of digital mental health products. Thus, it becomes clear that the evidence base is a highly complex and multifaceted aspect of digital mental health product quality. These considerations are even more critical given current national policy priorities. In the UK, the recently released 10-Year Health Plan advocates for digitally enabled care, including the integration of apps and self-management instruments into clinical pathways.^47^ In this context, the development and adoption of robust quality assurance frameworks and comprehensive evidence standards are fundamental to guarantee that digital mental health tools are safe, effective, and scalable within routine care.

Issues related to *data security and governance* are also highlighted as critical concerns, with both service users and healthcare professionals expressing apprehension about privacy breaches and inadequate protection of sensitive data.^48–50^ These concerns often hinder the adoption of digital products, as trust is crucial for their successful implementation. Moreover, professionals value platforms that allow better control over confidentiality, which help mitigate risks and facilitate secure remote assessments.^51^ At the same time, as shown by Alhammad and colleagues,^52^ users demand greater transparency and more robust data protection measures, with privacy features influencing their willingness to engage with such platforms. However, in an analysis of 61 mental health apps in 2017 and 56 apps in 2018, Parker et al.^53^ found that many lacked adequate privacy policies, and those that existed often did not meet basic standards for accessibility, readability, and comprehensiveness. Furthermore, these apps frequently sought access to significant amounts of user data without providing clear justification or a thorough explanation of how it would be used.

Unfortunately, existing frameworks and guidelines proposed for quality assessment of digital mental health tools fall short of addressing many of these aspects.^54^ The Mental Health App Usability Questionnaire (MAUQ),^55^ for example, focuses exclusively on usability. The American Psychiatric Association developed a comprehensive framework for health app evaluation^56^ which has been operationalised for public use through the Mhealth Index and Navigation Database (MIND).^57,58^ This tool was primarily designed for clinicians and consumers to evaluate apps, and so is limited in the aspects it can probe. For example, it does not assess whether service users were involved in the development process, as this information is often unavailable to end users,^58^ despite it being a crucial, yet often overlooked, design consideration.^59,60^ A similar limitation applied to PsyberGuide, a mental health app rating platform developed by OneMind,^61–63^ which is no longer operational.

### Aim of this study

This study seeks to develop a novel, comprehensive, and pragmatic quality assessment framework aimed specifically at digital mental health products within the parameters of the UK's healthcare system.

This newly developed framework aims to address critical gaps in the quality assurance landscape for digital mental health tools. Firstly, the framework acknowledges that most digital mental health tools operate outside formal healthcare systems and are therefore not subject to established standards or regulations. Secondly, the framework explicitly outlines what constitutes a high-quality tool in a mental health context. Thirdly, while existing frameworks often rely heavily on academic definitions of quality,^37^ the framework proposed here incorporates user input to ensure it delivers information that is both meaningful and relevant to consumers.

This work followed a thorough, iterative, user-driven, and system-conscious design process, aiming to create a quality assessment tool informed by research and stakeholder contributions, bringing together diverse stakeholders, including partners from the NHS, higher education institutions, small and medium-sized enterprises, and people with lived experience of mental health difficulties. Specifically, the research questions are:

1. What are the essential quality criteria for digital mental health tools?
2. How can these criteria be organised into a comprehensive quality assurance framework?
3. What are the key steps to effectively apply this framework in practice?

## Methods

### Design

This pragmatic study developed a novel quality assurance framework for digital mental health tools, using a sequential mixed methods design with an embedded qualitative component.^64,65^ Quantitative and qualitative survey data were collected simultaneously, followed by focus groups conducted after initial survey analysis to explore findings in greater depth.

Quantitative survey data were used to evaluate the perceived importance and credibility of quality principles for digital mental health tools across three stakeholder groups (1) mental health service users, family members, or members of the public; (2) healthcare workers or research professionals with clinical, academic, or managerial expertise; and (3) individuals working within technical, product, innovation, or commercial disciplines in digital health provision. For brevity, these groups are referred to as mental health service users, academic/healthcare professionals, and digital health provision experts, respectively.

Qualitative data from surveys and focus groups provided richer insights into these topics from a smaller sample representing the same three groups. This methodological approach was adopted because the quantitative survey enabled a broad analysis of the relative value of different proposed quality assurance elements, while qualitative data collection facilitated a more in-depth exploration of how well these elements capture stakeholder priorities and what additional factors they consider relevant to quality assurance and its implementation.

Reporting was informed by the GRAMMS (Good Reporting of A Mixed Methods Study) guidelines.^66^

### Participants

Participants were eligible to take part if they were aged 18 years or over, lived in the UK, and self-identified as one of three stakeholder groups (mental health service user, academic/healthcare professional, and digital health provision expert).

Participants were recruited through voluntary sampling. Advertisements for the online survey were disseminated across social media platforms such as LinkedIn, mailing lists, research opportunity boards, and relevant newsletters, offering a £10 incentive voucher. Participants for the focus groups were drawn from the survey sample, where they were given the opportunity to opt in for further participation.

The survey yielded 309 valid responses, with the majority of participants identifying as mental health service users, female, aged 21–30, and White, with most reporting no disabilities (see Table 1). Responses were analysed across the three predefined stakeholder groups to capture a range of perspectives relevant to the development of the quality assurance framework. Each group contributed distinct forms of expertise, including lived experience of using digital mental health tools, clinical and academic perspectives on safety and use, and practical experience in the development and implementation of digital technologies.

**Table 1.** The demographic characteristics of the survey participants (*N* = 309).

Demographic category	Sub-category	*n* (%)
Gender	Female	201 (65.05)
	Male	90 (29.13)
	Non-binary	14 (4.53)
	Trans-gender	2 (0.65)
	Other	1 (0.32)
	Prefer not to say	1 (0.32)
Age	18–20	45 (14.56)
	21–30	163 (52.75)
	31–40	66 (21.36)
	41–50	24 (7.77)
	51–60	7 (2.27)
	60+	4 (1.29)
Ethnicity	White	141 (45.63)
	Asian/Asian British	75 (24.27)
	Black/African/Caribbean/Black British	55 (17.80)
	Mixed/multiple ethnic groups	19 (6.15)
	Arab	5 (1.62)
	Other ethnic group	9 (2.91)
	Prefer not to say	5 (1.62)
Disability	No	230 (74.43)
	Yes	68 (22.01)
	Prefer not to say	11 (3.56)
Role	Mental health service user, family member, or member of the public	197 (63.75)
	Healthcare worker or research – clinical, academic, or managerial expert	105 (33.98)
	Technical, product, innovation or commercial discipline – expert in digital health provision	7 (2.27)

### Quantitative data collection and analysis

Quantitative data on stakeholder preferences and opinions were gathered through an online survey. The full survey instrument can be found in the Supplementary Materials. The survey was piloted internally with members of the project team (*n* = 10), including individuals with lived experience, clinical, academic, technical, and commercial expertise, to assess clarity, wording, structure, and alignment with the research aims. Minor refinements were made prior to full deployment.

As shown in the introduction, an initial review of the existing literature indicated that aspects related to UX/UI, data, and evidence is essential to the quality of digital mental health tools. The survey questions aimed to refine this initial framework and to determine how it can best be implemented. Participants were asked to rate items measuring the importance and credibility of quality principles for digital mental health tools. Example items included “The tool was developed collaboratively with people with lived experience”, “The tool is accessible and easy to use for its users”, “The content of the tool is based on established theories, published guidelines, and a scientific evidence base”, and “I would consider tools that have undergone this NHS-backed QA process as more trustworthy”.

The survey consisted of three main sections: stakeholders’ experiences with mental health digital tools, their priorities and opinions regarding the three identified framework domains, and their views on the feasibility and value of the assessment approach. The survey was hosted on Qualtrics, using a general anonymised link for completion. The survey was circulated via organisational newsletters, mailing lists, research opportunity boards, and social media, and it was open from November to December 2023. At the beginning of the survey, participants were provided with information about the study and had the opportunity to contact the researchers for further information before providing informed consent to participate. Following this, participants were asked to provide demographic information that directed them to tailored questions based on their stakeholder group. The survey took approximately 15–20 min to complete. Responses were manually verified by multiple members of the research team to confirm they were not bot-generated. This process involved checking for internal consistency, completeness, and any indicators of automated submissions. After verification, 309 usable responses were retained for further analysis. Quantitative data were analysed using Microsoft Excel, with descriptive statistics (e.g., means, standard deviations) computed to summarisè the results.

### Qualitative data collection and analysis

The qualitative data collection for this study included an online survey and focus groups aimed at understanding quality considerations for digital mental health tools. As previously described, the online survey, which collected 309 usable responses, featured open-ended questions allowing participants to elaborate on their experiences and expectations regarding digital mental health tools. For example, participants were asked to describe “What does it mean to you for a tool to be user-friendly?”, “What does it mean to you for a tool to be safe?”, and to provide additional feedback on priorities and considerations for digital mental health tools.

At the end of the survey, respondents were invited to indicate whether they would be interested in taking part in further qualitative discussions. In total, 154 respondents expressed interest, including 50 mental health service users (32.47%), 101 academic/healthcare professionals (65.58%), and three digital health provision experts (1.95%). The mean age of this subgroup of respondents was 29.49 years (SD = 8.38), and the majority identified as female (*n* = 83; 53.90%). Of these, 101 participants attended follow-up focus groups, comprising six online groups (conducted via Microsoft Teams), one online interview, and one in-person group held at the Denmark Hill campus of South London and Maudsley NHS Foundation Trust. No additional demographic data were collected at the focus group stage.

Focus groups were deliberately composed of mixed stakeholder groups. In order to minimise potential power dynamics and to foster open, collaborative dialogue, participants were not asked to disclose their lived experience or professional role. Importantly, the purpose of the focus groups was not to examine differences between groups, as these were already captured through the survey, but rather to provide further depth and context to the survey findings and support the development of a shared understanding of key issues across stakeholders.

The focus groups were facilitated by experts in psychology, clinical education, and digital health, and began with an overview of the project and incorporated open-ended questions to encourage in-depth discussion (e.g., “Can you describe your experience with digital mental health tools?”), supplemented by follow-up prompts to guide conversations towards specific quality markers (e.g., “What are the most important markers or considerations of quality for data management and privacy in these tools?”). The focus groups were audio-recorded and auto-transcribed. Transcripts were then reviewed, edited for accuracy, and de-identified. Braun and Clarke's thematic analysis^67–69^ guided the qualitative analysis process. The analysis was conducted in a deductive manner, with the initial domains derived from the literature guiding the thematic mapping of the data.

The researchers started the process by immersing themselves in the data, reading and re-reading survey responses, transcripts, and field notes to gain a deep understanding of the content. Using the predefined domains as a framework, the researchers systematically assigned codes to data segments that were relevant to each domain. For example, segments about ease of use were coded within the UX/UI domain with labels such as “navigation ease”, “functionality”, and “user preferences”. Similarly, data segments concerning privacy and security were coded under the “data” domain, while discussions on scientific validation were coded under the “evidence” domain.

The codes were then examined within each domain to determine how well they supported the predefined themes. This process ensured that the themes were representative of stakeholders’ experiences and perspectives. Any discrepancies or nuances found during analysis were discussed within the research team and used to refine the themes, therefore enhancing their coherence and validity. This deductive approach enabled the systematic validation of the initial domains (themes), confirming they effectively captured the most critical quality considerations for digital mental health tools. The final themes were integrated into the analysis to articulate what each theme represented and how it contributed to the understanding of quality in digital mental health tools, thereby informing the development and refinement of the quality assurance framework and assessment process.

We should note that data saturation was not treated as a predefined analytic goal in this study, reflecting its pragmatic, framework-development focus and mixed methods design. Instead, confidence in the robustness of the framework was established through triangulation across multiple sources, including quantitative survey findings, qualitative survey responses, focus groups, and the existing literature. Convergence across these data sources informed the refinement and validation of the framework domains and was further supported by iterative discussions between project team members and key stakeholders.

Reflexivity was considered throughout the qualitative analysis. The multidisciplinary research team brought expertise spanning digital mental health, clinical practice, education, and lived experience, which informed both data collection and interpretation. To mitigate the influence of individual assumptions, coding and theme development were discussed iteratively within the team, and interpretations were continually checked against the quantitative findings and the existing literature. This reflexive, team-based approach supported transparency and coherence in the development of the framework.

### Mixed-methods integration

Integration occurred at multiple points. First, open-ended survey responses were analysed alongside quantitative data to identify areas of convergence and divergence. Second, focus group discussions were structured to explore, clarify, and expand on survey findings. Finally, qualitative and quantitative results were triangulated by the project team in order to refine quality domains, operationalise assessment criteria, and inform the staged assessment process. Mixing methods enabled the study to capture both broad stakeholder priorities and nuanced perspectives, ensuring that the resulting framework reflected not only the relative importance of quality principles across stakeholder groups but also how these principles are interpreted and applied in practice. This approach allowed the framework to be both evidence-informed and user-centered, directly addressing the research aim of developing a practical quality assurance framework for digital mental health tools.

## Results

### Quantitative results

As part of the survey, participants were asked to rate the importance of various statements related to the initial domains on a scale from 1 (not important at all) to 5 (highly important). The purpose of this rating was to assess the perceived importance of these principles in establishing the effectiveness and credibility of digital mental health tools.

#### Effectiveness

Participants’ mean ratings for the effectiveness of digital mental health tools are presented in Table 2. Service users rated “Appropriate and relevant content” as the most important principle for establishing effectiveness, with a mean score of 4.30 (SD = 1.05). Healthcare professionals prioritised “Accessibility and ease of use” at 4.45 (SD = 0.89), while digital health experts rated both “Appropriate and relevant content” and “Continuous evaluation of UX and impact” equally high at 4.50 (SD = 0.55) and 4.50 (SD = 0.84), respectively.

**Table 2.** Mean ratings for the importance of principles in establishing the effectiveness of digital mental health tools across the initial domains and by stakeholder groups.

Initial domain	Statement theme	Mental health service user		Academic/healthcare professional		Digital health provision expert		Overall	
		*M*	SD	*M*	SD	*M*	SD	*M*	SD
*UX/UI*	Appropriate and relevant content	4.30	1.05	4.44	0.96	4.50	0.55	4.35	1.01
	Accessibility and ease of use	4.24	1.12	4.45	0.89	4.00	1.26	4.31	1.05
	Diversity of users considered	4.19	1.05	4.24	1.04	3.67	1.21	4.20	1.05
	Lived experience	4.05	1.14	4.24	0.92	4.00	1.55	4.11	1.08
*Data*	Risk of harm escalation	4.28	1.00	4.28	1.07	4.00	1.10	4.27	1.03
	Data safety governance	3.72	1.22	3.79	1.24	3.67	0.82	3.74	1.22
	Transparency regarding data collection	3.64	1.33	3.68	1.20	4.00	0.89	3.66	1.28
	Clear protocols for data usage	3.56	1.31	3.53	1.26	3.83	0.98	3.55	1.29
*Evidence*	Continuous review and updating	4.20	1.02	4.29	0.98	3.83	1.17	4.34	1.01
	Content based on scientific evidence	4.20	1.00	4.37	0.89	3.50	1.05	4.25	0.97
	Continuous evaluation of UX and impact	4.06	1.06	4.18	0.98	4.50	0.84	4.11	1.03
	Clearly defined pathway to change	4.02	1.07	4.23	0.92	3.50	0.84	4.08	1.02
	Research evidence base	3.81	1.17	3.95	1.09	3.67	1.37	3.85	1.15

#### Credibility

The participants also rated principles related to the credibility of digital mental health tools, as illustrated in Table 3. “Data safety governance” emerged as the most important principle for service users, with a mean rating of 4.41 (SD = 0.90). For healthcare professionals, “Transparency regarding data collection” was rated as the most important principle, with a mean of 4.54 (SD = 0.83). Digital health experts rated “Research Evidence base” the highest, achieving a mean of 4.83 (SD = 0.41).

**Table 3.** Mean ratings for the importance of principles in establishing the credibility of digital mental health tools across the initial domains and by stakeholder groups.

Initial domain	Statement theme	Mental health service user		Academic/healthcare professional		Digital health provision expert		Overall	
		*M*	SD	*M*	SD	*M*	SD	*M*	SD
*UX/UI*	Lived experience	4.09	1.10	4.27	1.04	4.33	0.82	4.16	1.08
	Appropriate and relevant content	4.11	1.00	4.19	0.95	4.50	0.84	4.15	0.98
	Diversity of users considered	4.11	1.12	4.12	1.08	4.33	0.82	4.12	1.10
	Accessibility and ease of use	3.62	1.23	3.72	1.18	3.33	1.03	3.65	1.20
*Data*	Data safety governance	4.41	0.90	4.48	0.96	4.50	0.84	4.44	0.92
	Transparency regarding data collection	4.34	1.08	4.54	0.83	4.00	1.55	4.40	1.02
	Risk of harm escalation	4.25	1.00	4.40	0.88	4.00	0.89	4.30	0.96
	Clear protocols for data usage	4.26	1.02	4.24	0.90	4.00	1.10	4.25	0.98
*Evidence*	Content based on scientific evidence	4.39	0.93	4.50	0.91	3.83	1.47	4.42	0.94
	Continuous review and updating	4.11	1.10	4.38	0.92	3.67	1.21	4.20	1.05
	Research evidence base	4.10	1.09	4.36	0.89	4.83	0.41	4.20	1.03
	Continuous evaluation of UX and impact	4.10	0.99	4.04	1.11	3.83	0.98	4.07	1.03
	Clearly defined pathway to change	3.91	1.06	4.12	0.90	3.50	1.52	3.97	1.02

#### Value of assessment process

Survey participants rated a set of statements tailored to their specific roles regarding the perceived value of a quality assessment process for digital mental health tools. Ratings were provided on a scale from 1 (Completely disagree) to 5 (Completely agree). These results are summarised in Table 4.

**Table 4.** Mean ratings for the perceived value of the quality assessment process for digital mental health tools by stakeholder groups.

Stakeholder group	Statement	*M* (SD)
*Mental health service user*	I would consider tools that have undergone this NHS-backed quality assurance process as more trustworthy	4.13 (1.07)
	I would be more likely to use a mental health tool that has undergone this NHS-backed quality assurance process over one that has not	4.09 (1.12)
	It is important that this quality assurance process is NHS-backed, rather than not	3.97 (1.06)
	Companies undergoing this quality assurance process for their tools are more appealing to me that those that do not	3.96 (1.06)
	This NHS-backed quality assurance process would help users receive better quality support	3.96 (0.97)
*Academic/healthcare professional*	I would feel more confident recommending tools that have undergone this NHS-backed quality assurance process	4.55 (0.72)
	I would consider apps that have undergone this NHS-backed quality assurance process more trustworthy	4.51 (0.79)
	This process will help improve and standardise the quality of digital mental health products	4.35 (0.87)
	I would use tools myself that have undergone this NHS-backed quality assurance process over ones that have not	4.27 (0.86)
*Digital health provision expert*	An NHS-backed quality assurance process would set me apart from my competitors for my clients/customers/payors (if different to end users)	4.14 (1.07)
	This NHS-backed quality assurance process will provide valuable feedback for the development of my tool	4.14 (0.90)
	I would be interested in going through an NHS-backed quality assurance process to obtain expert approval on my tool	4.00 (0.82)
	An NHS-backed quality assurance process would set me apart from my competitors for my end users	3.86 (0.90)

#### Time willing to spend on assessing evidence

Participants indicated the amount of time they were willing to spend assessing evidence for a digital mental health tool using a scale ranging from 0 to 100 h. On average, participants reported a willingness to spend 35.94 h (SD = 27.12) on this task.

### Qualitative findings

Table 5 summarises the qualitative findings derived from survey responses and focus groups. The analysis was guided by three initial domains derived from the literature, namely UX/UI, Data, and Evidence. These domains provided the structure for coding and mapping the qualitative data, which ultimately led to the refinement of the domains that constitute the basis of the MTAQ framework.

**Table 5.** Themes and sub-themes, illustrative quotes, and the initial and refined domains of the quality assurance framework.

Main theme	Sub-theme	Illustrative quotes
UX/UI and co-design	Usability and accessibility	• “Simple to navigate, clear instructions, easy to set up” • “It has to be intuitive and straightforward to navigate regardless of technological fluency” • “Making sure that it's accessible for people with different needs […] is the fonts accessible, you’re not using italics, you’re following digital accessibility. […] looking at the colours, colour of contrast, differentiations, different things like that, like using a digital accessibility guide when you’re building it.” • “If I talk about the surface structure of the tools, the some of the points which will attract me to use the tools […] will be the visual appearance. How the tools are constructed and easy navigation.”
	Co-design and lived experience involvement	• “What has been really helpful is having service user or people with lived experience involvement in developing it.”
	Cost and adoption considerations	• “Many of these apps are not actually free, they’re free, maybe for any period of time, but then after that you will still have to pay for them, which can be, you know, an inconvenience for many users.” • “The tool should have a lower barrier to entry, whether that barrier be monetary-wise, user ability-wise or accessibility-wise”.
Data privacy, security, and governance	Data control and consent	• “Control over personal data and settings, communication is transparent throughout […]” • “I would make sure that it even says that there's a deletion date. […] But after that deletion date, the data is no longer accessible.”
	Transparency in data handling	• “Privacy and safety of data has to be taken care of. I want to understand how my data will be used and what laws protect me.” • “If I can store my information, how will it be stored? Or what about the privacy policy? Will it be disclosed to other people or something like that?” • “If there's somebody externally also monitoring the system, so like if the anonymity was preserved, and I mean just privacy, maybe not anonymity and that that data collection was used in a beneficial way not to sell data to advertisers, it could in the future be beneficial.” • “So having a login and a password for your own if it's a more personalised digital mental health tool, you might want to do that so that no one can access if they were just on your phone or on your laptop so that you log in each time.”
Evidence-Based practices and ongoing evaluation	Evidence of effectiveness	• “If a tool is developed by academics/clinicians and is evidence based (i.e., there's information of how/why it works) then I would be more likely to trust it.” • “Based on evidence, based on the latest scientific understanding, tested and reviewed by a diverse set of people with lived experience.” • “Consistently providing accurate information, being transparent about its sources and methods, and maintaining a reputation for reliability.”
	Transparency and trust	• “There are now a lot of kind of wellbeing apps or apps that have brilliant visuals, but maybe the evidence base is not as strong. And then you’ve got apps which are a little bit more clinical looking that might be endorsed by a trust like an NHS Trust or an endorsed by your clinician.” • “I think when you see that something has got NHS affiliation or I guess you kind of assume one would assume that it's reached a certain quality standard, you know almost like a kite mark or something like that.” • “I think people like to know the evidence. […] I think often we forget that service users also know about science. They also know about data, you know, data collection and evidence, and and it's very important for someone to believe that the whatever what be it […] that that is going to work and is effective” • “And I think they work better, if I know, you know, what's the evidence base behind those of, you know, techniques or activities they are trying to promote. So have having more sort of evidence based or research based perspective and selling it as such.”

In addition, participants were asked about their needs, preferences, and expectations in relation to the assessment process, including their expectations for reviewing evidence, collaborating with stakeholders, and engaging in feedback and reporting; the type of evidence they consider essential to demonstrate the quality of mental health tools; and the support needed to conduct assessments. Grouped thematically, and as presented in Table 6, these insights informed the development of an assessment process which could guide the practical implementation of the quality assurance framework.

**Table 6.** Themes and sub-themes, illustrative quotes, and the preliminary stages of the assessment process.

Main theme	Sub-themes	Illustrative quotes	Preliminary assessment process stages
Transparency and clarity	Providing clear guidance, accessible information, privacy protection, and regulatory compliance	• “I would expect there to be some structure to the feedback and roles/expectations clearly defined.” • “A list of criteria to rank the tool against. Clear understanding of the aims/ goals of the tool.” • “So we try to create apps that will have different options like where you have a voice pad.”	Contracting
Engagement and professionalism	Delivering structured onboarding, managing expectations, and demonstrating the purpose and value of the assessment	• “Initial training, examples of what would and wouldn’t meet criteria, examples of good practice and ongoing assessor update training” • “Initial meeting with you and client to understand more about the tool and testing requirements; testing the tool first-hand; regular catch-ups to provide feedback and ask for more information if required.” • “Instead of going to the long way of writing, you know, up with, you know, much write ups”	Client onboarding
Tailoring and comprehensiveness	Personalising assessment to client requirements and ensuring a thorough review of relevant data	• “Initial training to understand exactly what I was supposed to be looking for and then reviews on the evidence gathered to ensure I was collecting the right type of information”	Review of assessment and client requirements
Participation and accessibility	Encouraging active client involvement, providing user-friendly channels for input, and flexible response options	• “Regular catch-ups to provide feedback and ask for more information if required.” • “I think that in the process of designing the show try the app should kind of be clear”	Client-led response and collation of evidence
Rigour and reliability	Structured, evidence-based review including scoring, clarifications, and verification to ensure assessment consistency	• “Clear guidelines of what is needed for the assessment (e.g., use of a rating scale for evaluating the evidence)” • “Relevant statistics about the tool performance (e.g., effectiveness in improving mental health, users’ engagement in the tool in terms of time, frequency etc.), reviewing evidence of the content included in the tool and decide if the content are deemed appropriate for inclusion, engage with key stakeholders including clients to review the facilitators and barriers of using the tool, address areas for improvement.” • “That would make something seem kind of trustworthy and reliable. I think yeah. Just having, you know, standards.”	Supplier-led review, clarifications and scoring
Quality assurance and recognition	Providing feedback on performance, recommending improvements, transparent scoring, publishing results, and certifying quality	• “Revising, updating and implementing the feedback/insights will be quite iterative and in collaboration with the various people involved.” • “Marking against assessment criteria, meetings discussing feedback and decisions made in developing the tool and offering ongoing feedback.” • “Also, I would say the ultimately the success of a tool is dictated by the users’ experience rather than specific criteria. So I think you could have criteria in terms of the provision of the service and the actual functioning of the tool. So the more technical parts.” • “I think probably if they know that it's, you know, in any way linked to some credible organisation or authorities or things like the NHS or […] the NIHR. So I think having that kind of link and official kind of approval or yeah from a trustworthy organisation that they are aware of.”	Outcome – provision of scores and feedback

### The Mental Health Technology Assessment of Quality (MTAQ) framework

**Figure 1.**
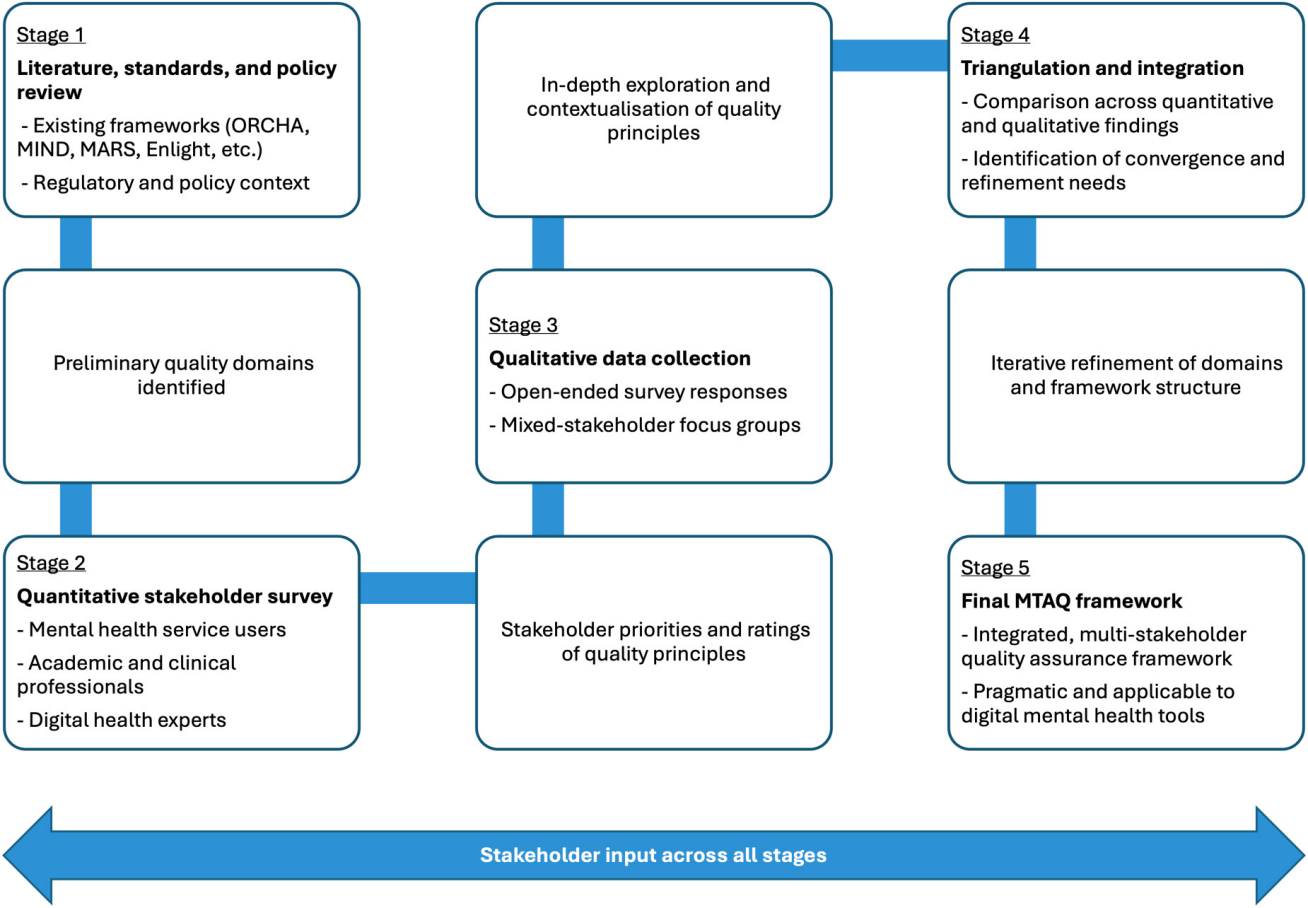
Staged, multi-stakeholder development process of the MTAQ framework.

The MTAQ framework was developed through a staged, iterative, and multi-stakeholder process, as illustrated in Figure 1.

The process began with an initial review of existing international standards, regulatory guidance, and evaluation frameworks relevant to digital health and digital mental health. This review informed the identification of preliminary quality domains and highlighted known limitations of existing approaches.

These preliminary domains were then examined and refined using quantitative and qualitative data collected from a broad range of stakeholders, as described previously. Survey items captured stakeholder priorities and perceptions regarding quality principles across domains such as UX, evidence, data governance, and safety. The qualitative component aimed to explore how stakeholders interpreted quality principles, how these were experienced in practice, and how they could be operationalised within a usable assessment and verification process.

Quantitative and qualitative findings were then triangulated to assess convergence, complementarity, and areas requiring refinement. This triangulation informed both the validation of the emerging quality domains and the design of a transparent, staged assessment process through which quality judgements could be made.

Integrating survey and focus group data allowed for a more nuanced understanding of stakeholder priorities. While quantitative results highlighted general trends in importance and credibility ratings, qualitative insights revealed how stakeholders interpreted these principles and identified additional considerations, which were not captured in the survey alone. This combination fed into the refinement of the framework, ensuring in this way the incorporation of both widespread priorities and richer contextual insights into the final MTAQ framework.

Throughout this process, regular input and feedback from stakeholders with lived experience of mental health difficulties, academic and clinical expertise, and technical and commercial experience in digital health contributed to iterative refinement.

**Figure 2.**
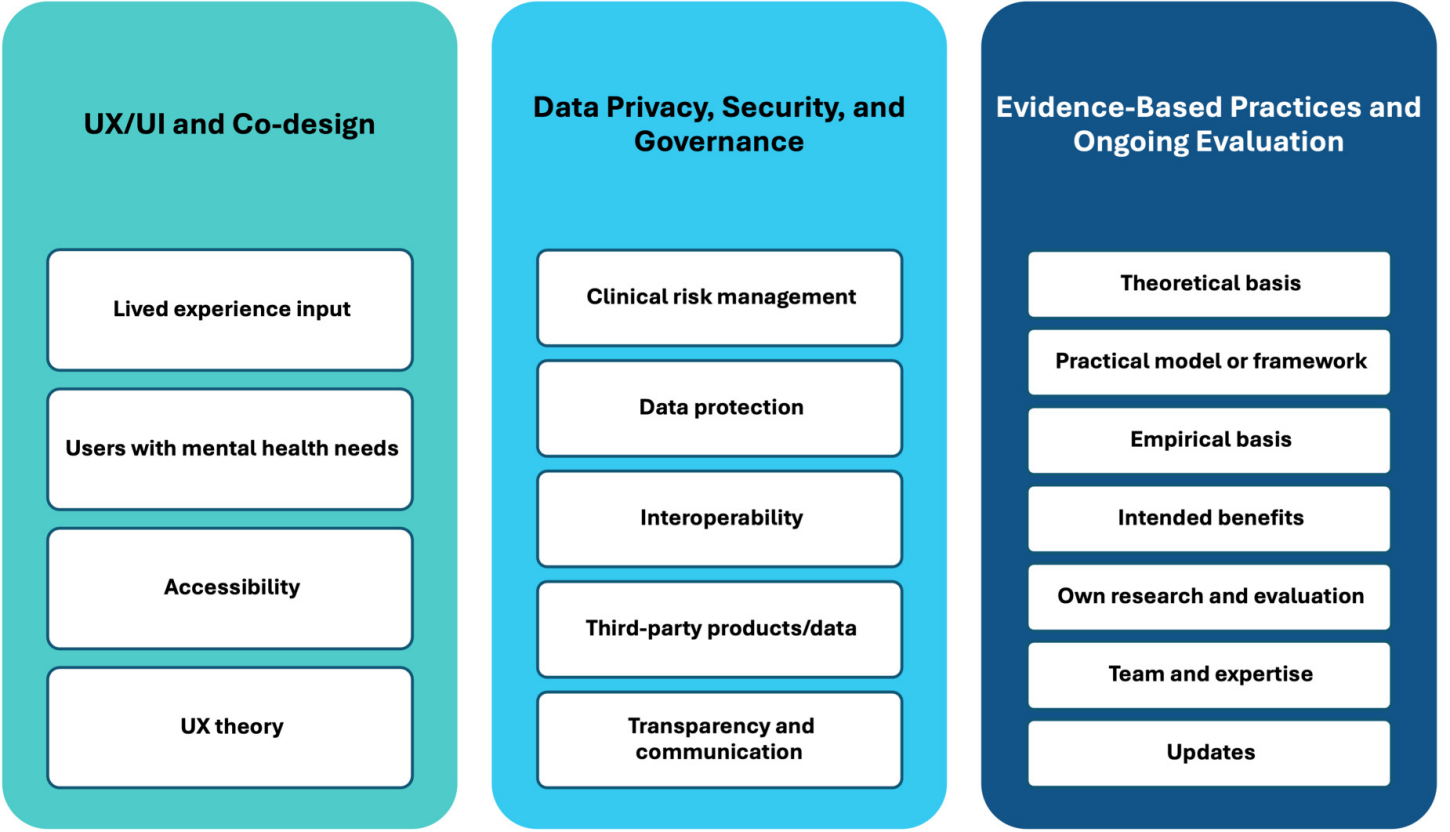
The MTAQ framework.

The final MTAQ framework reflects this integrated evidence base and multi-stakeholder perspective. This framework is structured around three core domains, which together define what quality means in the context of digital mental health tools (see Figure 2).

#### UX/UI and co-design

This domain emphasises the central role of user experience and co-design in the quality of digital mental health tools. Tools should be designed with a clear understanding of the needs, preferences, and constraints of people experiencing mental health difficulties, recognising that cognitive load, emotional state, motivation, and accessibility requirements may differ substantially from those associated with other types of digital health tools.

Paramount to this domain is the meaningful involvement of people with lived experience throughout the design, testing, and iterative refinement process, ensuring that tools are usable, acceptable, and aligned with real-world needs. Tools should accommodate a spectrum of mental health and wellbeing, including users experiencing acute distress, and reflect technology- or content-specific considerations.

Accessibility is another core consideration, encompassing navigation, visual design, feature access, and overall functionality, alongside language, cultural sensitivity, and inclusive design to mitigate barriers and promote equitable engagement. UX and co-design are further guided by established theoretical principles, drawing on relevant medical, psychological, and technological theories to underpin design choices.

Beyond usability, this domain encompasses engagement and sustained use, capturing whether tools provide meaningful learning resources, coping strategies, personalised recommendations, and feedback mechanisms, all of which influence trust, adoption, and the likelihood that tools will be used as intended.

#### Data privacy, security, and governance

This domain addresses the critical role of data management and governance in ensuring the quality of digital mental health tools. Tools must be transparent about the types of data collected, how these data are stored and processed, and the circumstances under which they may be shared. All information should be presented in clear and accessible language to support informed decision-making by users.

Data governance includes clinical risk management processes, such as maintaining formal logs and oversight by a designated safety officer, alongside broader organisational structures that ensure accountability. Data protection measures encompass consent procedures, risk assessments, mitigation strategies, robust policies, secure storage and processing, and user rights such as opt-out or deletion, with attention to data location.

Interoperability with healthcare systems or research institutions must comply with relevant standards while preserving strong data protection. For tools that incorporate third-party products or data, explicit safeguards and protection plans must be documented. Transparency and communication are central to this domain, ensuring that users can clearly understand how their data are used, how privacy is protected, and how any adverse experiences are recorded and addressed.

By integrating these technical, organisational, and communicative elements, the framework positions data privacy, security, and governance as foundational components of quality, directly influencing user trust, engagement, and the overall effectiveness of digital mental health tools.

#### Evidence-based practices and ongoing evaluation

The third domain focuses on the extent to which digital mental health tools are grounded in robust evidence and subject to ongoing evaluation. Tools should be informed by established medical, psychological, behavioural, or technological theories, with design and content decisions explicitly linked to these foundations. Developers should specify any models or frameworks guiding the tool, including how they have been applied or adapted in practice, and align their approach with recognised guidelines, such as those provided by NICE.

The empirical basis of a tool should be transparent, drawing on published scientific evidence and reports, and clearly demonstrating how this evidence has informed the design, functionality, and intended outcomes. Developers should articulate the intended benefits, expected outcomes, and potential side effects of the tool, while considering the broader context of use to avoid over-reliance on the digital intervention alone.

Ongoing evaluation is central to this domain. Developers should collect data on usability, engagement, adherence, and safety through both internal research and practical evaluations. Feedback should be actively sought from clinicians, academic researchers, and people with lived experience, and systematically incorporated into iterative updates to maintain and enhance the tool's quality and relevance over time.

The composition and expertise of the team responsible for development and evaluation are critical. Academic researchers, clinicians, and technical personnel should be involved to ensure that evidence is appropriately interpreted, integrated, and applied.

Finally, recognising the dynamic nature of digital mental health tools, the framework supports a pragmatic approach to evidence. While randomised controlled trials are valued, they may not always be feasible or appropriate. Complementary sources of evidence, including usability testing, engagement and adherence data, safety monitoring, clinical input, and stakeholder feedback, are essential. Mechanisms should also be in place to keep the evidence base current, incorporating emerging research, updated guidelines, and evolving best practice.

#### MTAQ assessment process

**Figure 3.**
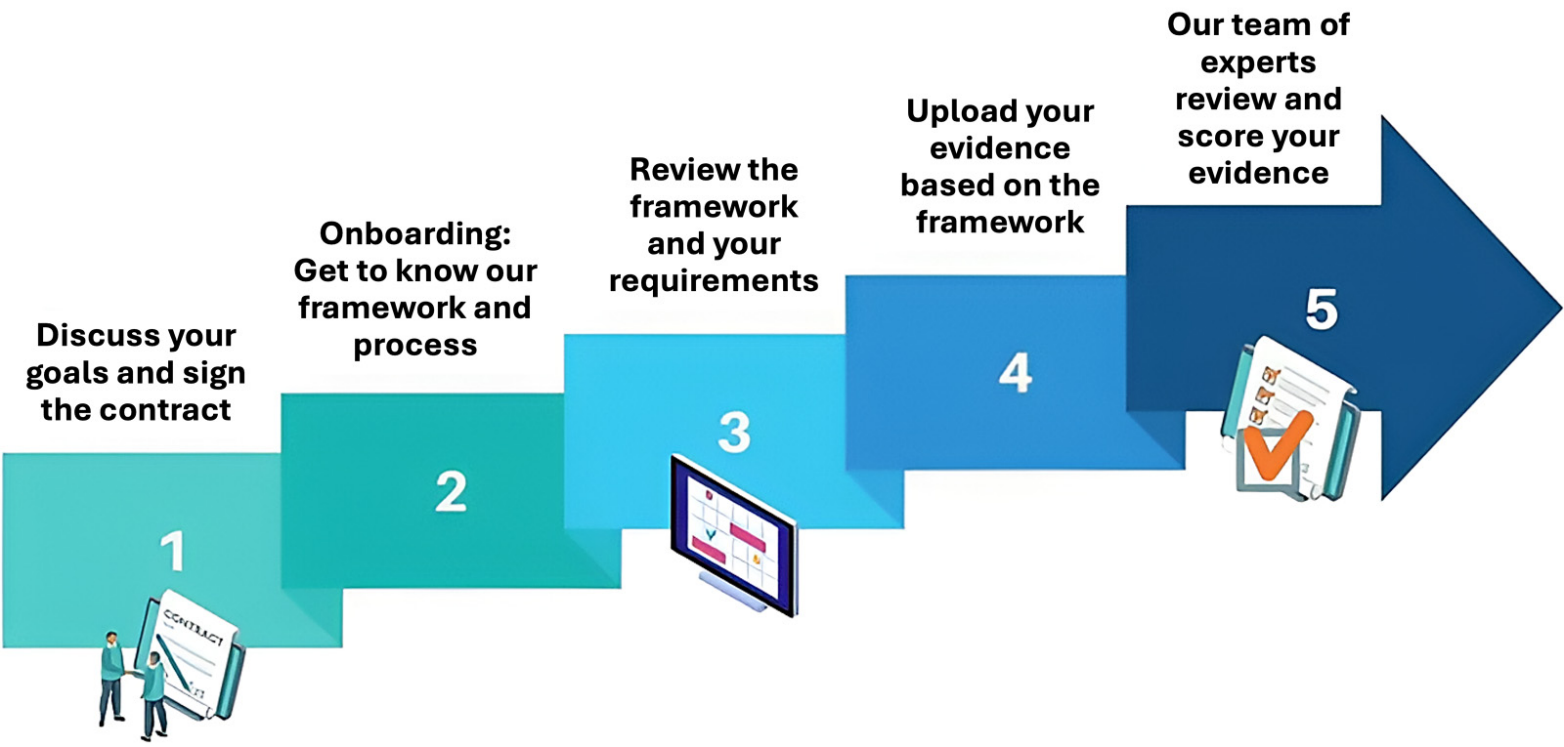
Operational workflow for evaluating digital tools using the MTAQ.

The MTAQ framework is operationalised through a structured assessment and verification process, refined into a sequence of five stages that guide tools from initial engagement through to quality recognition (see Figure 3). These stages include formal contracting and scoping, structured client onboarding, tailored review of assessment requirements, client-led collation of evidence, and an expert-led review involving clarification, scoring, and feedback.

Verification within MTAQ is therefore achieved through an iterative, evidence-informed process rather than a single evaluative step. Assessment outcomes are documented through transparent scoring and structured feedback, with recommendations for improvement where relevant. Tools that meet the required standards are awarded an MTAQ quality mark (kitemark), signalling that the product has undergone independent, structured assessment against the framework domains.

Digital mental health tools assessed against the MTAQ framework receive a published notification detailing the assessment outcome, completion date, product version, benchmarking information where applicable, and the required date for re-assessment. This approach positions the kitemark as both a signal of quality at a given point in time and mechanism for ongoing accountability as tools evolve.

## Discussion

This study presented the development of the MTAQ, which is a novel, comprehensive, and pragmatic framework and process for assessing the quality of digital mental health tools. The quantitative findings showed that different user groups prioritised various principles when evaluating the effectiveness and credibility of such tools. Importantly, in the context of digital mental health, ‘users’ or ‘consumers’ refers not only to service users but also to healthcare professionals and commissioners who prescribe, recommend, or make decisions about the use of these tools. Service users, for example, focused on the relevance and appropriateness of the content, while healthcare professionals emphasised accessibility and ease of use. On the other hand, digital health experts placed high value on continuous evaluation of UX and impact, as well as the evidence base supporting the tools. Furthermore, participants across all groups strongly supported the value of a quality assessment process. The qualitative findings provided key insights in relation to UX/UI, data, and evidence, which guided the refinement of the quality assurance framework. Participants stressed the importance of intuitive and accessible user interface, with specific calls for simpler navigation, lower barriers to access, and the integration of co-design approaches. Participants also advocated for clear communication about data collection, use, and storage, as well as giving users control over their personal data. More than that, there was strong support for tools being evidence-based, with participants preferring solutions backed by research and clear, transparent sources of information.

### Links with existing literature

The findings of this study align with and expand upon existing literature in the field of digital mental health. The emphasis placed on appropriate and relevant content resonates with previous studies that highlight the importance of tailoring digital interventions to individual needs.^41,70–72^ Appropriately tailored content not only facilitates engagement but also increases the perceived value and effectiveness of digital mental health tools.^73–75^ Taken together, these findings reinforce the need for participatory approaches in the development of digital mental health tools to better meet the diverse needs of users. The prioritisation of accessibility and ease of use echoes established principles of usability and inclusivity in human-computer interaction research.^76,77^ Several studies emphasise that tools designed with user-centred principles are more likely to foster sustained engagement, particularly for populations with varying levels of digital literacy.^78–80^ The call for intuitive design and lower barriers to access is further supported by evidence suggesting that complex interfaces and usability challenges act as key deterrents to adoption.^41,81^

The prioritisation of continuous evaluation aligns with frameworks advocating iterative and adaptive tool development. The growing literature on agile methodologies and user-centred design^77,82–85^ highlights the necessity of ongoing feedback loops to ensure that tools remain relevant, functional, and aligned with emerging user need. This is particularly important in a rapidly changing field, where tools must be adapted to technological advancements, shifting consumer expectations, and the diverse needs of different populations.^11^ At the same time, continuous evaluation is necessary to keep up with the evolving standards of evidence in digital mental health, which mirrors the preference for evidence-based tools that participants in this study expressed. Many apps and interventions struggle to meet or demonstrate compliance with these multifaceted standards,^9,16,42,46,86,87^ sometimes resulting in paradox where insufficient evidence hinders adoption, which in turn limits the possibility to generate new evidence.^87^ Furthermore, clinicians, who are often tasked with prescribing these tools, lack the resources, knowledge or confidence to adequately evaluate them,^88–90^ a challenge further complicated by the fragmented landscape of quality assurance and regulatory frameworks, as discussed before. Therefore, a more pragmatic, iterative approach to evaluation, one that prioritises adaptability, real-world testing, and ongoing feedback, is increasingly welcomed, with MTAQ being a strong example in this sense.

The findings pertaining to data-related aspects underscore the critical role played by transparency and control over the management of personal information in cultivating user trust, a theme well-established in the literature. For instance, past research has shown that users are more likely to trust and engage with digital health tools when they can exert autonomy over their data, including the ability to manage consent, delete information, and decide how data is shared, but concerns about data being sold to third parties or used beyond their knowledge or expectations might undermine the interaction with such tools.^91–94^ Notwithstanding the data privacy concerns, some participants recognised the potential benefits of data sharing, aligning with scholarship that suggests users are generally willing to do so when it enables personalised outcomes or supports the advancement of research.^91,92,95^ However, the willingness to share data hinges on clear and transparent communication about how this will be used, a need that may be even more prominent now given the expanding role of artificial intelligence and the ethical implications surrounding its use in digital (mental) health.^96–98^

### Comparison with other frameworks

Existing quality assessment frameworks, such as MIND and ORCHA, provide structured mechanisms to evaluate digital health tools. MIND operationalises a comprehensive set of criteria derived from the American Psychiatric Association app evaluation model, focusing on app functionality, evidence base, privacy and security, and data input/output.^57^ ORCHA's frameworks also use structured criteria, emphasising clinical safety, regulatory compliance, and usability to aid organisational procurement and certification.^26^ Both frameworks represent robust, standardised approaches to identify minimum standards and benchmark apps.

MTAQ differs from these frameworks in its explicit grounding in stakeholder perspectives and contextual priorities, having been developed specifically within the UK/NHS context. While MIND and ORCHA primarily evaluate tools against predefined technical and regulatory criteria, MTAQ incorporates qualitative insights from service users, healthcare professionals, and digital health experts to capture how key stakeholders value these criteria in practice, including trade-offs between usability, accessibility, and perceived evidence quality. This ensures that, in addition to compliance, assessment outcomes also reflect relevance and perceived credibility. Furthermore, MTAQ operationalises a stepwise assessment process, integrating contracting, onboarding, tailored data review, client-led evidence collation, and transparent scoring. This procedural clarity contrasts with the largely checklist-based evaluations of MIND and ORCHA, where stakeholder engagement and iterative feedback are less central. By bridging structured evaluation with qualitative stakeholder insight, MTAQ complements existing models, situating technical and regulatory criteria within a pragmatic, context-sensitive framework suitable for real-world implementation.

While MIND and ORCHA provide essential standardised metrics for evaluating digital health tools, MTAQ extends these approaches by incorporating stakeholder prioritisation, iterative assessment processes, and a focus on perceived trustworthiness and practical value. This positions MTAQ as both complementary and additive, addressing gaps in the literature related to user-informed quality assessment and real-world applicability.

### Strengths

This study directly addresses the lack of mental health-specific quality standards in existing digital health frameworks. By adopting a mixed-methods approach, the research incorporates perspectives from a wide range of stakeholders, including service users, healthcare professionals, academic researchers, and technical experts. The involvement of NHS clinical-academic professionals and people with lived experience of mental health difficulties strengthens the findings, confirming they reflect real-world priorities. The framework provides practical recommendations for developers and consumers, enabling safer selection of digital tools while also supporting improvements in mental health outcomes and product quality. Its evidence-informed and user-centred design, alongside the multidisciplinary approach, positions the research to bridge gaps between user needs and industry practices.

### Limitations

The study also has a number of methodological limitations. The framework has not yet been tested in real-world settings, and stakeholder representation was uneven, with relatively few digital health experts and a sample skewed towards younger, female participants, which may limit generalisability. Mental health service users were not asked about the specific services they had used, a decision made to reduce participant burden and protect confidentiality. The framework does not yet consider integration with healthcare systems, regional or cultural differences, or broader accessibility for general consumers. While the survey recorded broad stakeholder priorities, only a subset of respondents participated in focus groups, meaning some perspectives, particularly those of underrepresented groups, may not have been fully explored qualitatively. Because focus group discussions were guided by prior survey findings, there is a possibility that participants’ contributions were influenced by themes already identified, potentially limiting the emergence of entirely novel perspectives. Future research should validate the framework in diverse, real-world contexts, ensure broader demographic and stakeholder representation, examine its applicability across different service settings, and explore alternative qualitative approaches or consensus methods, such as a Delphi study, to further refine and operationalise the framework.

### Implications and recommendations

A number of key implications arise from this study that could inform the development and evaluation of digital mental health tools.

First, integrating co-design principles is essential to ensure that these tools are accessible, intuitive, and reflective of the diverse needs of users. By involving end-users in the design process, developers can create tools that are more responsive to specific user needs, improving both their usability and effectiveness. Second, digital mental health tools must promote transparency in data collection, usage, and storage. Clear communication about data governance policies allows users to retain control over personal information, which is crucial for building trust. Transparent data practices will foster confidence and make users more likely to use and recommend these tools. Third, incorporating mechanisms for regular updates and ongoing evaluation is necessary to make sure that digital tools remain relevant and effective over time. These tools must evolve in response to user feedback and advancements in research to remain useful. Regular evaluation and update allow tools to adapt to changing user needs and the latest evidence, contributing to their continued effectiveness.

Furthermore, the assessment process built around the MTAQ framework delineates a structured, practical approach for ensuring digital mental health tools meet the rigorous quality standards of MTAQ. The process consists of a series of stages, by the end of which compliant products receive the MTAQ verification mark. For users, the certification provides confidence that the tools they use have been thoroughly evaluated and meet established criteria for safety, efficacy, and UX. For developers, the process offers detailed feedback, highlighting areas for improvement and providing guidance on maintaining and enhancing quality over time. This also includes provisions for reaccreditation, certifying that tools continue to meet high standards and evolve in response to emerging evidence and regulatory changes.

## Conclusion

The MTAQ framework proposes a much-needed solution to the challenges surrounding the regulation and quality assurance of digital mental health tools. The growing prevalence of mental health difficulties, coupled with the increasing reliance on digital tools for mental health support, points to the urgent need for clear, robust, and evidence-based frameworks that guarantee the safety and efficacy of these tools. The three core domains of this novel, comprehensive, and pragmatic framework, (1) *UX/UI and Co-Design*, (2) *Data Privacy, Security, and Governance*, and (3) *Evidence-Based Practices and Ongoing Evaluation*, are directly aligned with the critical quality concerns identified in the literature and feedback from service users, healthcare professionals, academic researchers, and technical experts. MTAQ was developed with the UK context in mind, including alignment with NHS systems and stakeholder priorities, and is intended to provide a structured, practice-focused process for evaluating digital mental health tools. In doing so, it supports developers and consumers in identifying high-quality products and addresses the study aim of creating a framework that is both evidence-informed and user-relevant. Further real-world validations and adaptation to local contexts are necessary for its broader implementation.

## Supplemental Material

sj-docx-1-dhj-10.1177_20552076261428307 - Supplemental material for The Mental Health Technology Assessment of Quality (MTAQ): Development of a novel quality assurance framework for digital mental health tools Supplemental material, sj-docx-1-dhj-10.1177_20552076261428307 for The Mental Health Technology Assessment of Quality (MTAQ): Development of a novel quality assurance framework for digital mental health tools by Chris Attoe, Raul Szekely, Marta Ortega Vega, Emma Browne, Rebecca Thomas, Cho Wing Tiffany Kwan, Polina Altchouler, Anushka De, Nina Hariharan, Geraldine Clay, Sarah Odoi, Sean Cross and Andrew Doe in DIGITAL HEALTH
