# Supplementary material for: The Mental Health Technology Assessment of Quality (MTAQ): Development of a novel quality assurance framework for digital mental health tools
Source: Digit Health. 2026 Feb 24;12:20552076261428307. doi: 10.1177/20552076261428307 (PMC12932889; doi:10.1177/20552076261428307)
Supplement: sj-docx-1-dhj-10.1177_20552076261428307 - Supplemental material for The Mental Health Technology Assessment of Quality (MTAQ): Development of a novel quality assurance framework for digital mental health tools [file sj-docx-1-dhj-10.1177_20552076261428307.docx]

**Good Reporting of A Mixed Methods Study (GRAMMS) checklist**

O'Cathain A, Murphy E, Nicholl J. The quality of mixed methods studies in health services research. J Health Serv Res Policy. 2008;13: 92-98.

| **Guideline** | **Section (page)** |
| --- | --- |
| Describe the justification for using a mixed methods approach to the research question | Methods – Design (p.11)   - “This pragmatic study developed a novel quality assurance framework for digital mental health tools, using a sequential mixed methods design with an embedded qualitative component.” - “This methodological approach was adopted because the quantitative survey enabled a broad analysis of the relative value of different proposed quality assurance elements, while qualitative data collection facilitated a more in-depth exploration of how well these elements capture stakeholder priorities and what additional factors they consider relevant to quality assurance and its implementation.” |
| Describe the design in terms of the purpose, priority and sequence of methods | Methods – Design (p.11)   - “Using a sequential mixed methods design with an embedded qualitative component.” - “Quantitative and qualitative survey data were collected simultaneously, followed by focus groups conducted after initial survey analysis to explore findings in greater depth.” - “Quantitative survey data were used to evaluate the perceived importance and credibility of quality principles…” - “Qualitative data from surveys and focus groups provided richer insights into these topics from a smaller sample…” |
| Describe each method in terms of sampling, data collection and analysis | Methods – Participants (p.12)   - “Participants were recruited through voluntary sampling.” - “Advertisements for the online survey were disseminated across social media platforms such as LinkedIn, mailing lists, research opportunity boards, and relevant newsletters.” - “Participants for the focus groups were drawn from the survey sample, where they were given the opportunity to opt in for further participation.”   Methods – Quantitative Data Collection and Analysis (p.15)   - “Quantitative data were analysed using Microsoft Excel, with descriptive statistics (e.g., means, standard deviations) computed to summarise the results.”   Methods – Qualitative Data Collection and Analysis (pp.16-17)   - “Braun and Clarke’s thematic analysis guided the qualitative analysis process.” - “The analysis was conducted in a deductive manner, with the initial domains derived from the literature guiding the thematic mapping of the data.” |
| Describe where integration has occurred, how it has occurred and who has participated in it | Methods – Mixed-Methods Integration (p.18)   - “Integration occurred at multiple points.” - “First, open-ended survey responses were analysed alongside quantitative data to identify areas of convergence and divergence.” - “Second, focus group discussions were structured to explore, clarify, and expand on survey findings.” - “Finally, qualitative and quantitative results were triangulated by the project team in order to refine quality domains, operationalise assessment criteria, and inform the staged assessment process.” - “Triangulation across multiple sources, including quantitative survey findings, qualitative survey responses, focus groups, and the existing literature.” - “Iterative discussions between project team members and key stakeholders.” |
| Describe any limitation of one method associated with the present of the other method | Discussion – Limitations (pp.38-39)   - “Because focus group discussions were guided by prior survey findings, there is a possibility that participants’ contributions were influenced by themes already identified, potentially limiting the emergence of entirely novel perspectives.” - “While the survey recorded broad stakeholder priorities, only a subset of respondents participated in focus groups, meaning some perspectives… may not have been fully explored qualitatively.” |
| Describe any insights gained from mixing or integrating methods | Results – The Mental health Technology Assessment of Quality (MTAQ) Framework (p.28)   - “While quantitative results highlighted general trends in importance and credibility ratings, qualitative insights revealed how stakeholders interpreted these principles and identified additional considerations, which were not captured in the survey alone.” - “This combination fed into the refinement of the framework, ensuring the incorporation of both widespread priorities and richer contextual insights into the final MTAQ framework.”   Methods – Mixed-Methods Integration (p.18)   - “Mixing methods enabled the study to capture both broad stakeholder priorities and nuanced perspectives…” |
